# Computer-assisted, high-frequency, hospital-wide point prevalence surveys of hospital-acquired infections in a tertiary care hospital, the Netherlands, 2013 to 2014

**DOI:** 10.2807/1560-7917.ES.2019.24.13.1800177

**Published:** 2019-03-28

**Authors:** H Roel A Streefkerk, Sten P Willemsen, Conrad P van der Hoeven, Margreet C Vos, Roel PAJ Verkooijen, Henri A Verbrugh

**Affiliations:** 1 Department of Medical Microbiology and Infectious Diseases, Erasmus University Medical Centre, Rotterdam, the Netherlands; 2 Albert Schweitzer Hospital, Dordrecht, the Netherlands; 3 Beatrix hospital/Rivas Group, Gorinchem, the Netherlands; 4 Regional Laboratory for Medical Microbiology, Dordrecht, the Netherlands; 5 Department of Biostatistics, Erasmus University Medical Centre, Rotterdam, the Netherlands; 6 Department of Medical Microbiology, University Medical Centre, Groningen, the Netherlands

**Keywords:** automated surveillance, epidemiology, healthcare-associated infections, infection control, statistics, surveillance

## Abstract

**Background:**

Surveillance of hospital-acquired infections (HAI) often relies on point prevalence surveys (PPS) to detect major deviations in the occurrence of HAI, supplemented with incidence measurements when more detailed information is needed. In a 1,320-bed university medical centre in the Netherlands, we evaluated an electronically assisted surveillance system based on frequently performed computer-assisted PPS (CAPPS).

**Aim:**

The primary goals were to evaluate the performance of this method to detect trends and to determine how adjustments in the frequency with which the CAPPS are performed would affect this performance. A secondary goal was to evaluate the performance of the algorithm (nosocomial infection index (Nii)) used.

**Methods:**

We analysed the data of 77 hospital-wide PPS, performed over a 2-year period (2013 and 2014) and including 25,056 patients.

**Results:**

Six trends with statistical significance were detected. The probability to detect such trends rapidly decreased when PPS are performed at a lower frequency. The Nii and its dynamics strongly correlated with the presence of HAI.

**Conclusion:**

Performing computer-assisted, high frequency hospital-wide PPS, is a feasible method that will detect even subtle changes in HAI prevalence over time.

## Introduction

Currently, many hospitals rely for their surveillance of hospital-acquired infections (HAI) on a standardised hospital-wide point prevalence survey (PPS) to obtain information about the prevalence and types of HAI in their hospital. In the Netherlands, a national PPS is offered twice a year by the Dutch national HAI surveillance organisation PREventie van ZIEkenhuisinfecties door Surveillance (PREZIES), where all PPS data are collated, resulting in useful nationwide information about the prevalence of HAI [1]. However, for the hospital itself, the information gained from two PPS a year is limited, because at this frequency, PPS reveal little about the true variance in infection rates. Also the number of patients per department and per medical discipline included in each PPS is commonly too low to detect meaningful trends with any statistical power. To address these issues, one could perform multiple serial PPS at a much higher frequency, e.g. every month or even every week. However, hospital-wide PPS typically are labour-intensive if performed manually and require the input of trained infection control practitioners. Consequently, to our knowledge, there is only one study that presents serial hospital-wide PPS performed over a year or longer [2]. In our university hospital, we validated an algorithm (nosocomial infection index (Nii)) and implemented software to assist our infection control team to perform hospital-wide PPS more efficiently, as we previously reported [3,4]. The method was subsequently validated in a general hospital setting [5]. In the present manuscript, we primarily aimed to show how the results of frequently performed, hospital-wide computer-assisted PPS (CAPPS) could be converted into information about trends by type of HAI and by ward or medical specialty level, and how the frequency of measurements would affect the trend analyses. As a secondary aim, to guide the future development of computer-assisted surveillance systems, we studied the dynamics of the patients’ Nii scores in relation to the occurrence of HAI. We also studied the performance of the Nii algorithm in terms of predictive value and efficiency.

## Methods

### Setting

The Erasmus University Medical Centre includes a 1,320-bed tertiary care hospital in Rotterdam, the Netherlands. It provides a full range of services including a children’s hospital, an oncology centre, transplantation unit and a thorax centre. It also has an emergency department and functions as a regional trauma centre.

Included in this observational, descriptive and analytical cross-sectional study are the results of a series of 77 consecutive, hospital-wide CAPPS of HAI, of which 51 were performed in the year 2013 (once a week) and 26 in the year 2014 (every other week). Eligible for inclusion in these CAPPS were all patients, except those admitted to the day treatment center, to the department of psychiatry and those coming for haemodialysis. Patients who were admitted on the survey day were also excluded. However, these patients were included in the following CAPPS in case they still stayed at the hospital that long. Besides the above inclusion criteria for PPS by the Dutch national HAI surveillance network PREZIES, the infection control professional (ICP) used PREZIES criteria and definitions to assess the patients for the presence of HAI [6].

### Computer-assisted point prevalence survey and nosocomial infection index

An in-house developed algorithm-driven and previously validated software programme was used to perform the CAPPS [3-5]. A full description of this method is available in the Supplement. Key is an automated algorithm that calculates a score (the Nii) for every day of stay of every patient hospitalised. It is based on selected predictive parameters that are present on or in a time period of 7 days before the date of the prevalence survey (C-reactive protein (CRP), leukocyte count, microbiological examinations and antibiotic prescriptions, parameters that are digitally available in most hospital information systems). Each parameter present is awarded points which add up to the Nii. The cut-off value of 8 points was determined retrospectively from the results of two PPS conducted by the ICP among a surgical population in 2007. Here we analysed different sets of digitally available parameters and variable points per parameter were analysed. Based on receiver operator curve analysis, the cut-off value of 8 points had the optimal results for sensitivity and specificity (data not shown). Based on the Nii, the point prevalence population was stratified into two groups; those with an Nii score ≥ 8 on the survey day were reviewed by the ICP, whereas those with an Nii score < 8 were not reviewed by the ICP but were considered not to have an HAI on the day of the PPS. For each PPS, we documented the result per patient, i.e. HAI present or not, based on the ICP’s assessment or by default when the Nii score was < 8.

### Trend analysis for the computer-assisted point prevalence surveys

Firstly, we plotted the point prevalence rate of each of the 77 hospital-wide PPS. We then fitted a logistic regression model on the patient data, in which we modelled the probability of an infection using a function of time that was linear on the log odds scale. To examine the underlying pattern of the prevalence in more detail and to check the adequacy of the model using a linear trend, we also estimated a model using a regression spline of time. In this model, we allowed for more complex patterns over time. By using a penalty, the pattern was shrunk back to a linear trend when this complexity was not needed [7]. This analysis was repeated for medical disciplines with a higher risk of surgical site infections, lower respiratory infections, urinary tract infections and bloodstream infections. To address the problem of an inflated probability of a type I error caused by multiple testing, we applied a Benjamini-Hochberg correction. This multiplicity correction ensured that the overall chance of a type I error did not increase alpha (which was set at 0.05).

To elucidate the effect of the frequency of surveys on the ability to detect trends in HAI, we performed simulation-based power calculations for the trends with p < 0.05 and calculated the probability (expressed as the power P (1-beta)) to detect these trends when the CAPPS were performed weekly or once every 2, 4 or 6 weeks.

### Analysis of the performance of the nosocomial infection index

Secondly, we performed an analysis of the relationship between the Nii score on a given PPS date (Thursdays) and the fraction of patients diagnosed to have an HAI by the ICP, and the positive predictive value of an Nii score was calculated.

Since for each patient, Nii scores were present for each day of stay (not only for the point prevalence date) it was possible to track changes in individuals’ Nii scores during their stay and thus become informed about the in-hospital dynamics of patients’ Nii scores. The daily median and interquartile range (IQR) of the Nii scores were subsequently calculated for three categories of patients: (i) patients who had an Nii score of ≥ 8, who were reviewed by the ICPs and found to have an HAI (category 1); (ii) patients who had an Nii of ≥ 8, who were likewise reviewed but for whom review resulted in the decision that the patient did not have an HAI (category 2); and (iii) patients who did not have an Nii score of ≥ 8 on any point prevalence date during their hospital stay(s) and who were therefore not reviewed since they were considered not to have an HAI (category 3). A quantile regression analysis was performed on the dynamics of the Nii scores of category 1 and 2 patients. The median and IQR of the lengths of stay of each category of patients and the median and IQR of the interval between the day of admission and the day of an HAI diagnosis were also calculated and their relationship was studied.

All analyses were done in the ‘R’ software environment (version 3.4.1, see www.r-project.org) [8].

### Ethical statement

Approval by the ethical committee was not needed; in this non-interventional study we evaluated aggregated, anonymised data and results that were available through routine surveillance practice. 

## Results

For all patients in the hospital in the 2-year period 2013 and 2014, an Nii score was calculated and stored in the study database for each day of their stay. In 77 consecutive hospital-wide point prevalence populations, 25,056 (62%) patients were included. The patients who were excluded consisted of three groups: (i) patients who did not meet the clinical inclusion criteria, for example the haemodialysis patients, (ii) patients whose admission period did not include a Thursday (PPS day), i.e. admitted after a Thursday and discharged before the following Thursday, and (iii) patients for whom Thursday was the admission date and who were discharged before the next Thursday.

In total, 51,900 Nii scores on point prevalence dates were generated, which values ranging from −20 to +55. Overall, 15,051 of 51,900 (29%) Nii scores were at or above the cut-off value of 8 [3,4]. Review by the ICP of these 15,051 Nii scores resulted in the ascertainment of 2,810 HAI, i.e. 112 HAI per 1,000 patients included in the surveys.

### Trend analysis for the computer-assisted point prevalence surveys

**Figure 1:**
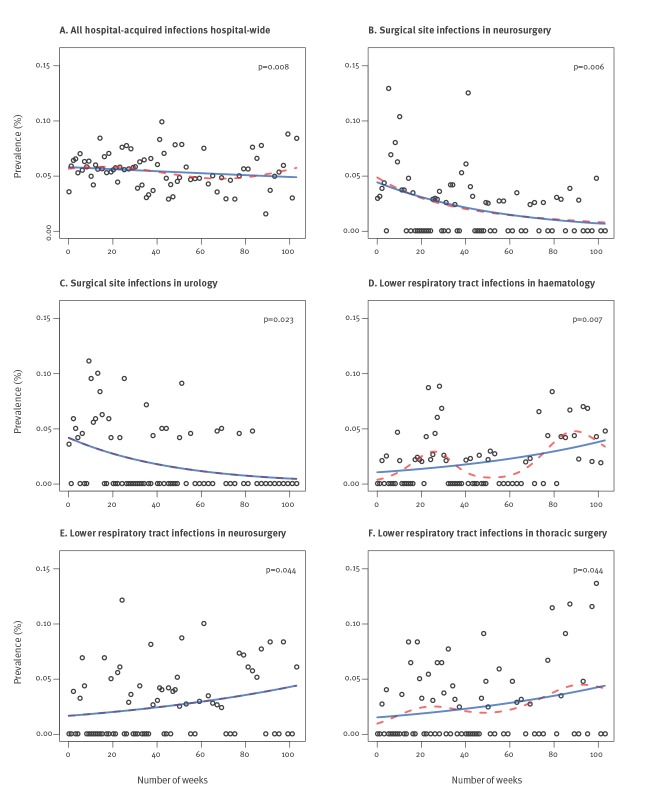
Healthcare-acquired infections prevalence rates obtained in 77 serial computer-assisted point prevalence surveys, Netherlands, 2013–2014 (n = 51,900)

Analysis of the results of the 77 CAPPS showed that HAI prevalence rates varied considerably from survey to survey (Figure 1A). The median hospital-wide HAI prevalence rate was 0.056 (IQR: 0.046–0.065). Trend analysis of the hospital-wide overall HAI prevalence rate showed a slowly decreasing trend (p = 0.008). Trend analysis of surgical site infections among patients admitted to the neurosurgery and urology department showed decreasing trends that were subtle but significant (p = 0.006 and p = 0.023, respectively) (Figure 1B, 1C), while the rate of lower respiratory tract infections increased in the departments of neurosurgery (p = 0.044), haematology (p = 0.007) and thoracic surgery (p = 0.044), (Figure 1D, 1E, 1F). We did not detect any significant trends in the prevalence rate of bloodstream infections and urinary tract infections in any department.

Open circles: individual prevalence rates; solid blue line: prevalence rates as modelled by logistic regression with a linear trend (on the scale of the odds ratios); red dashed line: logistic regression where we allowed for non-linearity by using a regression spline.

**Figure 2:**
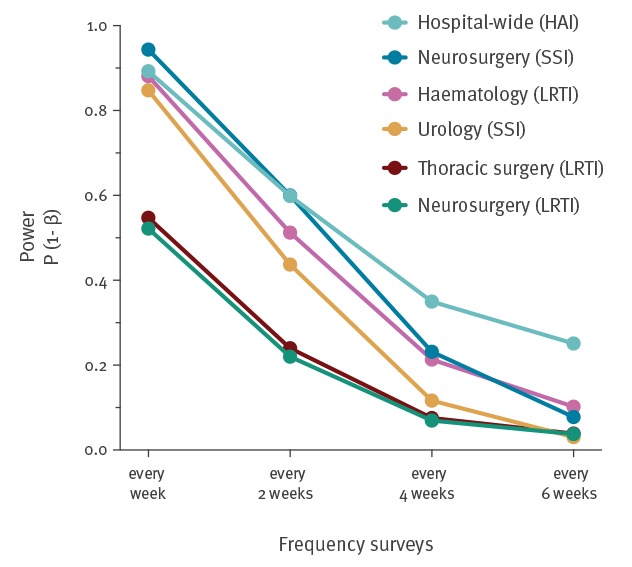
Effect of survey frequency on its power to detect significant trends in the occurrence of indicated types of hospital-acquired infections at department level, Netherlands, 2013–2014 (n = 51,900)

HAI: hospital-acquired infection; LRTI: lower respiratory tract infection; SSI: surgical site infection.

The probability of detecting trends decreased rapidly when the CAPPS were performed less frequently than once every week or every 2 weeks; at frequencies of once every 4 or 6 weeks, the probability of detecting such trends became very low (< 0.26 and < 0.10, respectively) (Figure 2). For example, the probability of detecting the truly decreasing trend in surgical site infections (SSI) in the department of neurosurgery fell from > 90% to < 10% if the frequency of the prevalence surveys was reduced from (bi)weekly to once every 6 weeks (Figure 2).

### Performance of the nosocomial infection index

**Figure 3:**
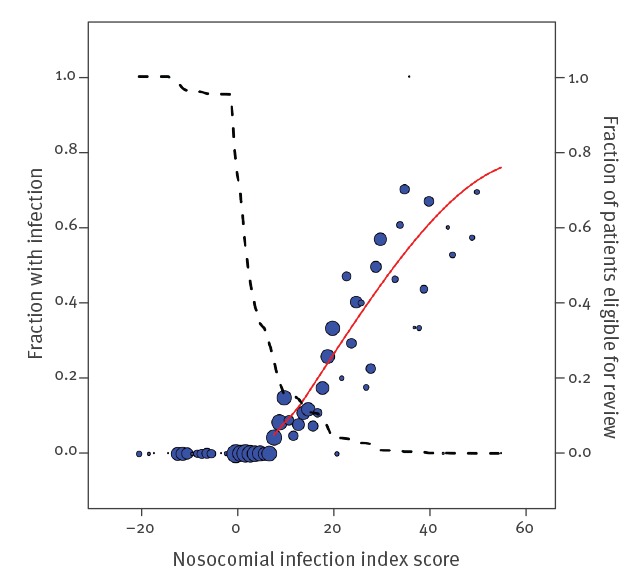
Fraction of patients with a hospital-acquired infections, by nosocomial infection index score on a survey date, Netherlands, 2013–2014 (n = 51,900)

HAI: hospital-acquired infection; Nii: nosocomial infection index.

The patients’ daily Nii scores were subsequently analysed and related to the presence or absence of an HAI on a given prevalence survey date. Figure 3 presents a plot of all 51,900 Nii scores in which the size of each bubble reflects the number of patients having a particular Nii score. The fraction (15,051 of 51,900 (29%)) of patients remaining eligible for on-screen review quickly diminishes when Nii scores rise. For Nii scores ≥ 8, there was a correlation between the Nii score and the fraction of patients diagnosed to have an HAI. Of the patients with an Nii score of 9, ca 10% had an HAI. This percentage increased to more than 60% when the Nii score was ≥ 40 (Figure 3). Overall, the positive predictive value of an Nii score ≥ 8 was 19% (2,810/15,051).

The bubble’s size reflects the number of patients with the indicated Nii score, the larger the bubble the more patients with that particular score. The dashed line represents the fraction of patients that at a given Nii score remained eligible for review. The red line represents the correlation between the Nii score and the fraction of patients with that score diagnosed to have an HAI using a local polynomial regression (Lowess) [8]. Patients with an Nii score < 8 were automatically marked HAI-negative.

**Figure 4:**
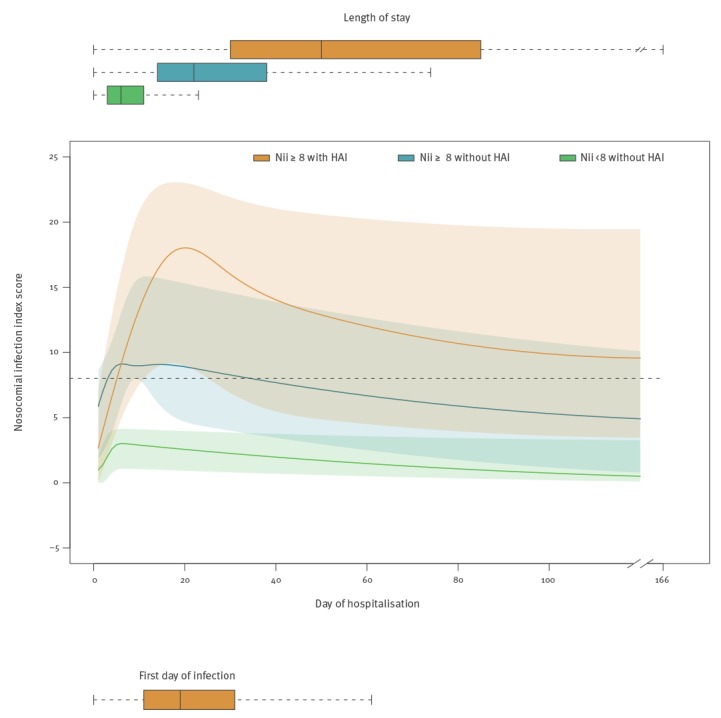
Dynamics of the nosocomial infection index score of patients with and without a hospital acquired infection, Netherlands, 2013–2014 (n = 51,900)

HAI: hospital-acquired infection; IQR: interquartile range; Nii: nosocomial infection index; PPS: point prevalence survey.

The Nii scores for each patient for each day of stay (i.e. not only the Nii scores on point prevalence dates) and their medians with IQR are presented in Figure 4. In total, 443,240 Nii scores were included in this analysis. Interestingly, category 1 patients ascertained to have an HAI had lower Nii scores on the day of their admission to hospital compared with category 2 patients who were suspected and therefore reviewed but found not to have an HAI (mean: 2.42 vs 4.36; median: 2 vs 3, p < 0.001). However, the Nii scores of patients developing HAI rose steeply towards a median peak of 18 at a median of 21 days after their admission; these dynamics differed from those of category 2 patients whose Nii peaked at a median of 9 at a median of 6 days after admission. The median day on which a diagnosis of HAI could be established (19 days) fell a few days before the peak in the median Nii score curve of the patients developing an HAI (Figure 4). Interestingly, the median Nii score in category 1 patients remained ≥ 8 during their admission, while it decreased below 8 in category 2 patients (Figure 4). Quantile regression analysis showed a significant difference between the dynamics of the Nii scores of category 1 and 2 patients. The Nii dynamics of patients who were categorised as not having an HAI because their Nii remained < 8 were also significantly different from those observed in category 1 and 2 patients (p < 0.001) (Figure 4). Clearly, patients who developed an HAI had a longer length of stay than patients who did not have such an infection (Figure 4).

Coloured lines and shaded areas represent median and IQR, respectively, of the Nii score per day of stay for each category of patients. Red: patients who developed an HAI (category 1); blue: patients who had an Nii ≥ 8 on a PPS date, but review by the ICP showed the patient did not have an HAI (category 2); green: patients whose Nii stayed < 8 on all PPS dates during their hospital stay (category 3). The three box plots above the figure depict the length of stay (median and IQR) of each category of patients. The single box plot below the figure depicts the interval (median and IQR) between the day of admission and the day of HAI diagnosis. The whiskers represent minimum and maximum values limited to 1.5 times the interquartile range.

## Discussion

This study shows that subtle but significant trends in HAI prevalence can be detected using serial (bi)weekly PPS. Based on a series of 77 consecutive computer-assisted hospital-wide surveys performed over a 2-year period, we obtained detailed insight into the variation of HAI prevalence in large a university medical centre. This was made possible by implementing a validated algorithm-driven and computer-assisted survey method by which hospital-wide PPS can be performed efficiently and accurately [3,4].

Considerable variation in the hospital-wide and the ward- and discipline-specific point prevalence rates from one survey to another became evident in our study. This observation supports the idea that, if only performed once or twice a year, PPS provide little information regarding the HAI epidemiology in a given hospital, except for quantifying the momentary burden of disease caused by HAI. Similar to our findings, two earlier serial prevalence studies reported mean HAI rates of 7.76% (with the lowest prevalence in this series being 2.44%, the highest 30.43%) and 5.42% (lowest prevalence 1.9%, highest prevalence 8.4%). A formula to convert point prevalence rates into incidence rates has been proposed in the past [2,9-11], but some authors noted that incidence rates cannot be derived reliably from PPS data [12,13].

In the 2-year study period, the overall hospital-wide HAI prevalence showed a decreasing trend (p = 0.008). However, we realise that trends in overall HAI prevalence are composed of up- and downward trends of different types of infections in different types of hospital departments surveyed, which may well result in a levelling effect when trends are compounded into a single hospital-wide HAI trend. Therefore, we also analysed trends of HAI separately per type of infection and per medical specialty where the risk of their occurrence was highest. Through this analysis, another five subtle, but significant, trends were detected. Importantly, by simulation-based power calculations, we showed that the probability that such trends will be detected would have declined, e.g. from more than 0.9 to less than 0.1, if CAPPS had been performed only once every 6 weeks instead of weekly. Thus, the frequency of PPS is key in detecting HAI trends with this method, an a priori plausible hypothesis that has been quantified and is supported by this study.

Predictably, the higher a patient’s Nii score was on the prevalence date the higher the probability that the patient had an HAI. This correlation makes the Nii on any given day a good predictor for the presence or absence of an HAI, although its maximum positive predictive value remained under 80%. Enhancing the discriminating power of the Nii algorithm would improve the efficiency of the CAPPS even further. The significant differences in the dynamics of Nii scores between patients with and without an HAI can possibly be used in future adaptations of the algorithm to improve its discriminatory power. Among patients suspected to have an HAI because of a daily Nii score exceeding 8, there is a sizable group of patients that do not have an HAI but have admission diagnoses, e.g. community-acquired infection or non-infectious inflammatory syndromes. Most of them enter the hospital with a somewhat elevated Nii score but this score does not rise much further during their hospital stay. Further analysis of this cohort of patients may therefore allow us to adjust our algorithm and make it more discriminatory.

PPS are generally believed to be more cost-effective than incidence surveys, and this is supported by our experience in this 1,320-bed university hospital where weekly hospital-wide CAPPS required the dedication of approximately one full-time equivalent ICP [3-5]. Using this algorithm-based method, 71% of the patients were automatically excluded from review by the ICP and marked negative. Likewise, Broderick et al. reported a 67% decrease in workload by excluding patients from detailed review [14]. Sensitivity and specificity of previously published comparable computerised algorithms for hospital-wide PPS ranged between 0.78 and 0.966 and between 0.73 and 0.98, respectively. The reported time gained by these automated survey methods ranged from 30% to over 99% [15-20]. As stated before, the negative predictive value of our Nii (i.e. a score < 8) was very high (> 99%). In our study, the assessment by the ICP was facilitated by an automatically generated on-screen timeline of all relevant data and a workflow management system (see Supplement). It is this on-screen decision support system that introduced an extra efficiency gain compared to manual bedside or (electronic) patient record review of the algorithm-selected patients. Likewise, Du et al. reported that their hospital-wide CAPPS required only 3.5 h using a visual time-series chart, while their traditional survey method needed 756 h per survey [18].

The potential value of detecting trends by frequently performed CAPPS needs to be interpreted in the context of some limitations. Firstly, this was an observational study in a period where major new interventions to reduce HAI were not implemented in this hospital. Clinicians were not aware of this new surveillance method and wards were not visited specifically for this study. Therefore, the few detected trends cannot readily be ascribed to a specific intervention or explained otherwise. Secondly, although the surveys were computer-assisted, the workload was still appreciable (±1 full time equivalent) and, facing restricted manpower, we therefore decided to perform CAPPS every other week in the second year of the study period instead of weekly as originally planned. We realise that doing so will have reduced the probability to detect HAI trends over the whole observation period and we may have missed more subtle trends than those that were detected. During the study, the data were not analysed, nor was information derived from the surveys disseminated to those who can take action in the departments. Still, in conclusion, a computer-assisted high frequency PPS-based surveillance system for HAI that is hospital-wide and includes all types of HAI is very informative regarding the HAI prevalence and its variance across the hospital and over time. It is highly sensitive to changes in HAI epidemiology, it will pick up even subtle trends in the occurrence of HAI that may be relevant and otherwise remain undetected. This information can be used to target interventions, and it is likely that the effects of these targeted interventions on HAI rates can be ascertained at an early stage by using this surveillance system.

## Supplementary Data

128900 Supplement
