# Supplementary material for: Computer-assisted, high-frequency, hospital-wide point prevalence surveys of hospital-acquired infections in a tertiary care hospital, the Netherlands, 2013 to 2014
Source: Euro Surveill. 2019 Mar 28;24(13):1800177. doi: 10.2807/1560-7917.ES.2019.24.13.1800177 (PMC6446511; doi:10.2807/1560-7917.ES.2019.24.13.1800177)
Supplement: Supplement [file 18-00177_Supplement_STREEFKERK.pdf]

"This supplementary material is hosted by Eurosurveillance as supporting information alongside the article Computer-assisted, high-frequency, hospital-wide point prevalence surveys of hospital-acquired infections in a tertiary care hospital, the Netherlands, 2013 to 2014 on behalf of the authors who remain responsible for the accuracy and appropriateness of the content. The same standards for ethics, copyright, attributions and permissions as for the article apply. Eurosurveillance is not responsible for the maintenance of any links or email addresses provided therein."

How a CAPPS is performed

In this document a quick overview is given of the e-surveillance software that was developed at the Erasmus MC to support the infection control professionals to perform a two-year series of hospital-wide computer-assisted point prevalence surveys (CAPPS) for hospital-acquired infections in an university hospital, as well as the validation in another hospital setting.

Most important requirement for the e-surveillance software was to maximally support the infection control professionals with each step of a computer-assisted point-prevalence survey, including multi-person review and conflict resolution.

Each step of this process, the workflow (figure 1), and its functional requirements are described.

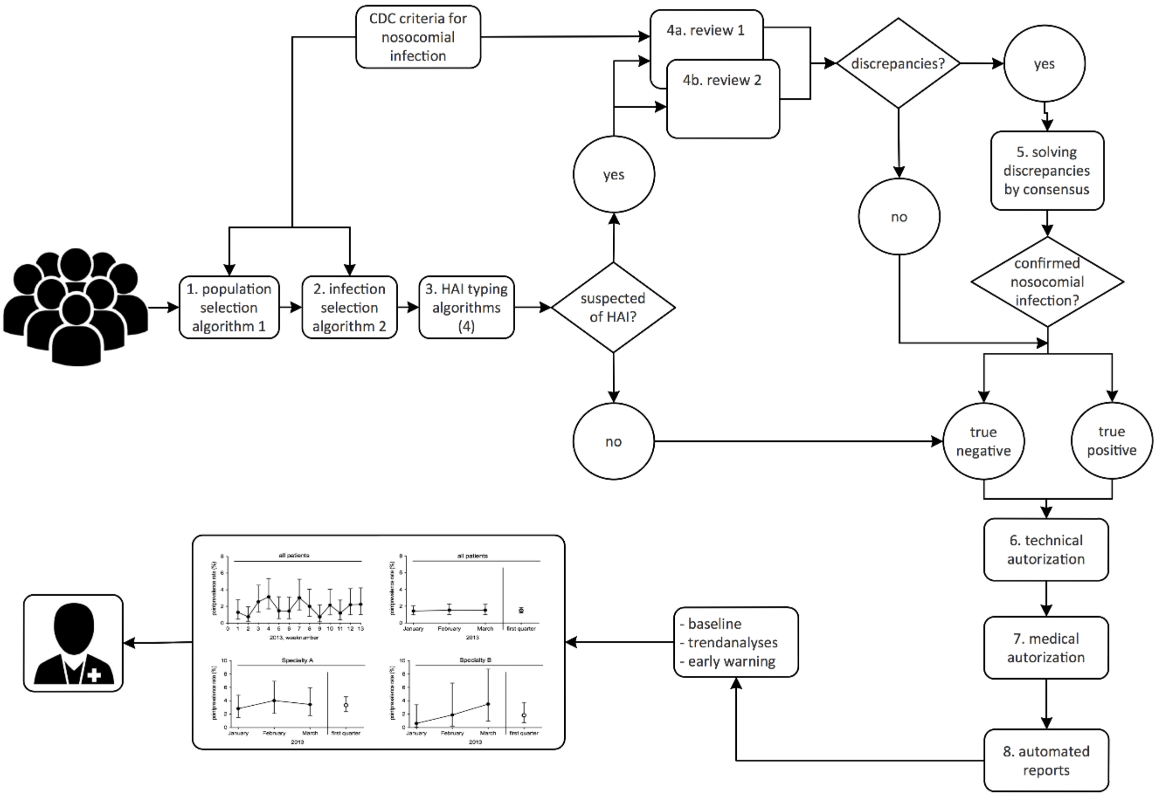

Figure 1: Workflow of a CAPPS. Steps 1-7 are described in more detail in this document.

Workflow

To perform a hospital-wide computer-assisted point-prevalence survey several steps are taken, which are described below and schematically presented in figure 1.

Step 1. Automatically determining the point prevalence population

By PREZIES definition a point-prevalence population (PPP) includes all admitted patients, except those admitted to day-care, to the department of psychiatry and those coming for hemodialysis. Patients who are admitted on the survey day are also excluded.

To support an automatically generated PPP the e-surveillance software needs to have access to census data within the hospital information system. This is done by a so called 'Admission, discharge and transform' (ADT) interface and every moment there is a change in patient location or medical specialty the information is updated in the e-surveillance software's data warehouse (figure 2).

On the point-prevalence day the PPP is generated using expert rules in a rule engine based on the PREZIES definitions.

Step 2-3. Algorithm based selection of patients for review by the infection control professionals

An important efficacy step in the workflow of an electronically assisted surveillance system is to automatically differentiate between those patients who are likely to suffer from a hospital acquired infection and those who do not. This is done using an algorithm-based selection mechanism that uses diagnostic and clinical variables from different sources in the hospital's information system.

To fully automate this step the variables have to be systematically extracted from their different sources and stored in the e-surveillance data warehouse (figure 2), so they can be employed by the rule-engine to execute the algorithm that results in a Nosocomial Infection Index (Nii) -score, on which the selection is based.

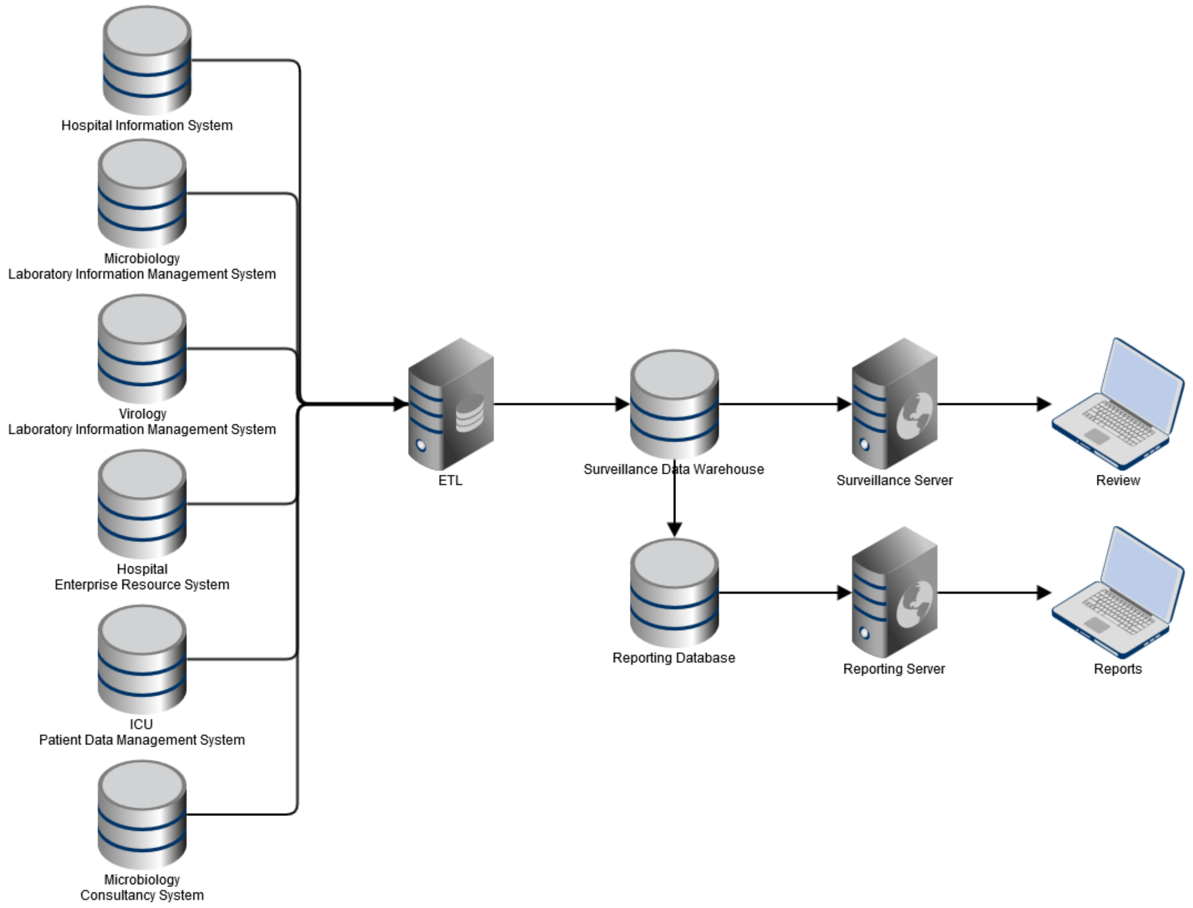

Figure 2: Surveillance Data Extraction

Calculating the Nii-score

The e-surveillance software uses a rule based system for calculating the NII-score. The underlying technology is Windows Workflow Foundation (WWF) version 3. With this technology it is possible to visually compose blocks of code and create rules in a rule editor (figure 3). Rules consists of conditions, if-actions and then-actions. If-actions fire when the condition is true, else actions when the conditions is false. An example of a rule is:

If maximum temperature in the past 1 days >= 38 degrees
Then add 5 points to the HAI property score

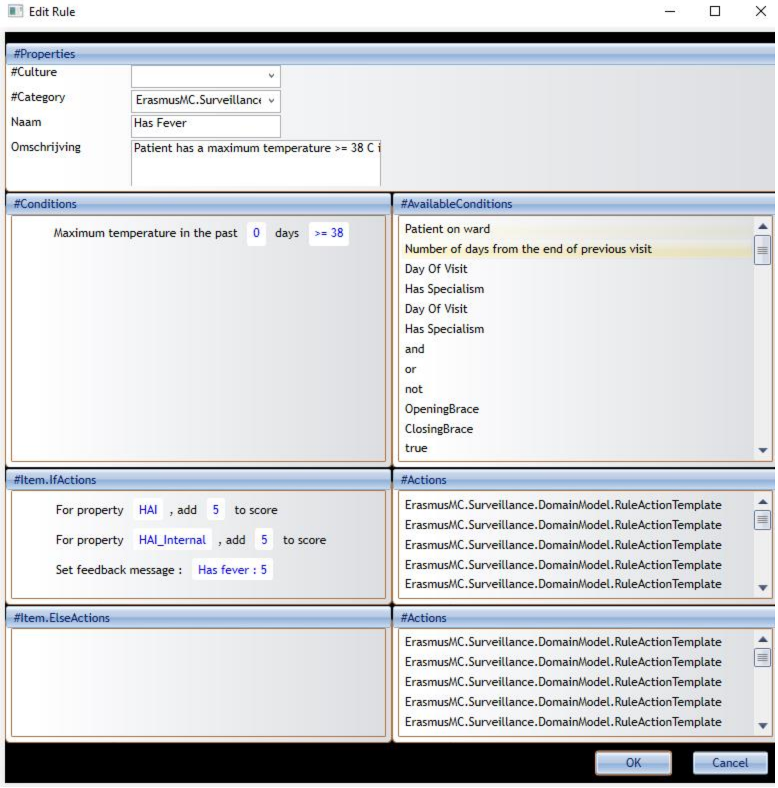

Figure 3: Rule editor

There is a variety of different rule categories for creating logic pertaining to the patient location, medication, laboratory results etc. The rules are composed into rulesets and can have a priority within the ruleset. The rules are processed against the patient data and when a condition is met the score is altered. The HAI (2.0) ruleset consists of the rules shown in figure 4

HAI 2.0	Hospital acquired infection score	DayContext	
#Name	#Description	#RuleCategory	#Enabled
Has CRP Test	Checks if the patient has a C-Reacti	Laboratory Tests	☑
CRP value > 48	Checks if the patient has a C-Reacti	Laboratory Tests	☑
Has Leukocyte Test	Checks if the patient has a Leukocy	Laboratory Tests	☑
Leucocyte value < 4 or > 12	Checks if the patient has a leucocyt	Laboratory Tests	☑
Is On Antibiotics Evaluation	Patient is on antibiotics	Property Evaluation	☑
Has Microbiology Specimen in pas	Checks if the patient has a microbic	Laboratory Tests	☑
Has BSI	Patient has a bloodstream infection	Property Evaluation	☑
Has UTI	Patient has a urinary tract infection	Property Evaluation	☑
Has LRTI	Patient has a lower respiratory trac	Property Evaluation	☑
Has SSI	Patient has a surgical site infection	Property Evaluation	☑
Clos Diff Toxine	Patient has clostridium difficile toxi	Laboratory Tests	☑
Metronidazol	Patient has Metronidazol antibiogra	Medication	☑
Has Fever	Patient has a maximum temperatur	Vital Signs	☑
On admission	Patient has had an infection from a	Property Evaluation	☑

Figure 4: HAI 2.0 rule set

Rule sets are organized into workflows. The HAI workflow is shown in figure 4. Every day a scheduled process creates the hospital population of the previous day based on the available location data. For each patient in the population the relevant patient data is gathered. Next the algorithm filters out prophylactic antibiotics and computes the scores for the different HAI-categories like bloodstream infection and surgical site infection. Based on the outcome of these subtype scores and a number of

other rules the overall HAI score is computed and stored in the data warehouse. All the rules that fire with the corresponding scores are also stored in the data warehouse.

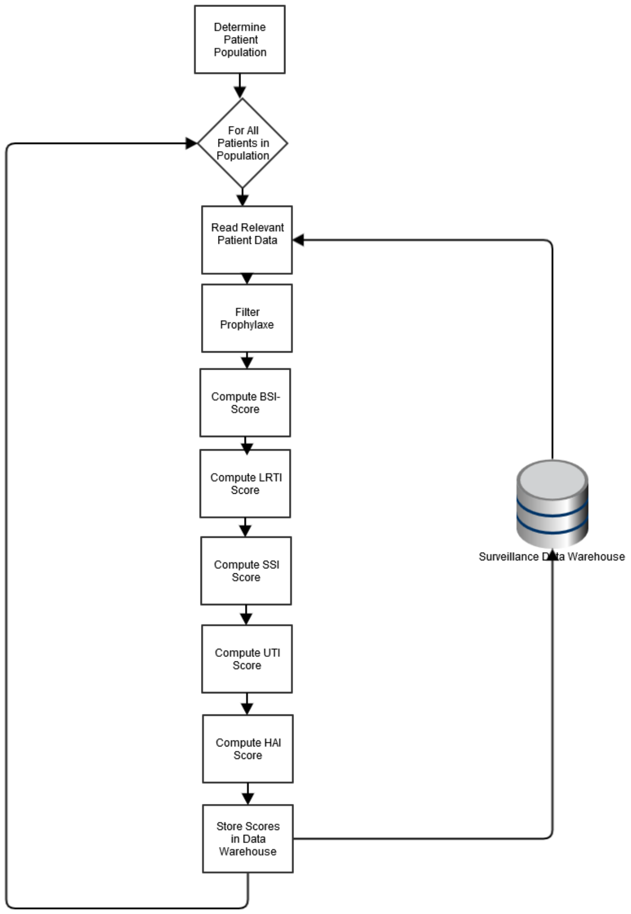

Figure 3: HAI 2.0 algorithm workflow

Selection of patient with Nii score ≥ 8 for review

All patients with a NII score < 8 are automatically removed from the population of patients that need to be reviewed. The remaining population of patients with a NII score ≥ 8 are selected for review and presented a survey population in the dashboard (figure 4)

#Name	Status	Gedaan	Te doen	Ziekenhuispoj	Geëxcludeerd	Prevalentiepo	HAI	BSI	SSI	SSI_Adm	UTI	LRTI	Anders	Laatst Gewijzigd
Aa	Aa	=	=	=	=	=	=	=	=	=	=	=	=	Aa
10-08-2017	Ready	0	0	927	0	927	0	0	0	0	0	0	0	
27-07-2017	Ready	0	0	918	0	918	0	0	0	0	0	0	0	
13-07-2017	Ready	0	0	947	0	947	0	0	0	0	0	0	0	
15-06-2017	Ready	0	0	919	0	919	0	0	0	0	0	0	0	
01-06-2017	Ready	0	0	942	0	942	0	0	0	0	0	0	0	
18-05-2017	Ready	0	0	916	0	916	0	0	0	0	0	0	0	
04-05-2017	Ready	0	0	926	0	926	0	0	0	0	0	0	0	
20-04-2017	Ready	0	0	934	0	934	0	0	0	0	0	0	0	
06-04-2017	Ready	0	0	951	0	951	0	0	0	0	0	0	0	
23-03-2017	Ready	0	0	916	0	916	0	0	0	0	0	0	0	
09-03-2017	Ready	0	0	697	0	697	0	0	0	0	0	0	0	
02-03-2017	In Progress	0	161	763	0	763	0	0	0	0	0	0	0	
23-02-2017	Ready	0	0	923	0	923	0	0	0	0	0	0	0	
09-02-2017	Ready	0	0	932	0	932	0	0	0	0	0	0	0	
12-01-2017	Ready	0	0	909	0	909	0	0	0	0	0	0	0	
22-12-2016	Ready	0	0	902	0	902	0	0	0	0	0	0	0	
08-12-2016	Ready	0	0	449	0	449	0	0	0	0	0	0	0	
24-11-2016	Ready	0	0	540	0	540	0	0	0	0	0	0	0	
10-11-2016	Ready	0	0	921	0	921	0	0	0	0	0	0	0	
27-10-2016	In Progress	0	183	903	0	903	0	0	0	0	0	0	0	
13-10-2016	In Progress	0	176	952	0	952	0	0	0	0	0	0	0	
29-09-2016	In Progress	0	194	913	0	913	0	0	0	0	0	0	0	
15-09-2016	In Progress	0	160	718	0	718	0	0	0	0	0	0	0	
01-09-2016	In Progress	0	184	875	0	875	0	0	0	0	0	0	0	
18-08-2016	In Progress	0	174	825	0	825	0	0	0	0	0	0	0	
10-07-2014	In Progress	161	1	946	0	946	0	0	0	0	0	0	0	347700
26-06-2014	In Progress	0	183	906	0	906	0	0	0	0	0	0	0	
12-06-2014	In Progress	0	140	890	0	890	0	0	0	0	0	0	0	
2012-10	In Progress	0	183	569	0	569	0	0	0	0	0	0	0	
Charlotte	In Progress	0	1123	1123	0	1123	0	0	0	0	0	0	0	
2011-10	In Progress	0	128	563	0	563	0	0	0	0	0	0	0	

Figure 4: Dashboard

Step 4. Review of selected patients by two infection control professionals

After logging on using a personal user account the infection control professional is presented with the dashboard with survey populations ready for review. There is a separate dashboard for first and second review indicating which populations need to be reviewed, how much patients within each population need to be reviewed and who is currently reviewing the population if the review is in progress. Two infection control professionals will independently review the same survey population.

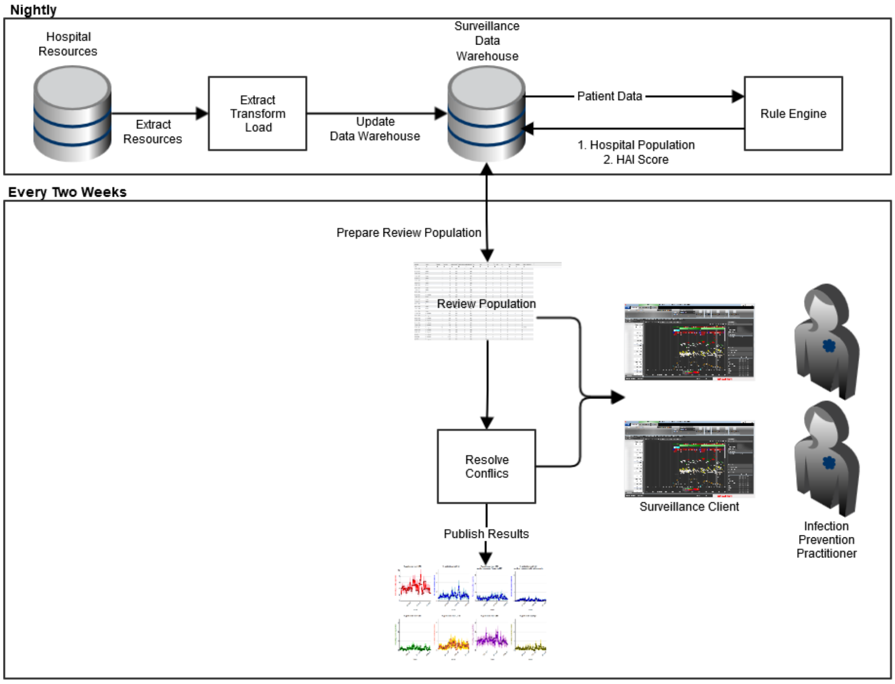

(fig 5)

Patient timeline

The infection prevention professional uses the e-surveillance client to evaluate and score the algorithm-selected patients. In the client all relevant information about the patient is conveniently presented in a timeline (each day is represented by one column, from left to right). Clinical reports can be displayed in full by resting the mouse cursor on a clinical report icon (B, D, I, E, O). The red color of a clinical report indicates that a keyword like “fever” is detected in the report.

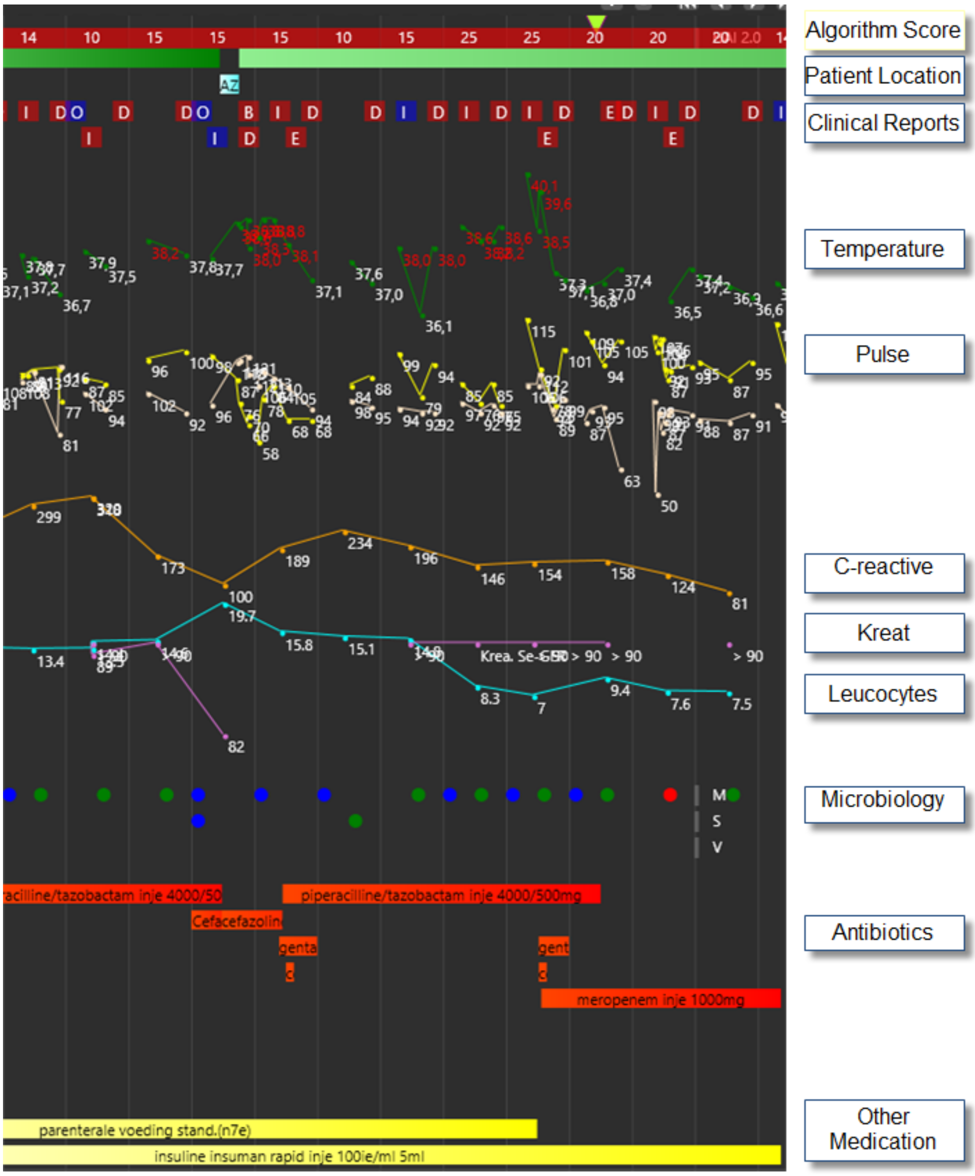

Figure 1: Patient timeline showing all relevant information

Details regarding the detected microorganisms can be found by resting the mouse on a positive (red) microbiology result. The names of the organisms are shown with the kind of specimen they were detected in and the susceptibility can be retrieved.

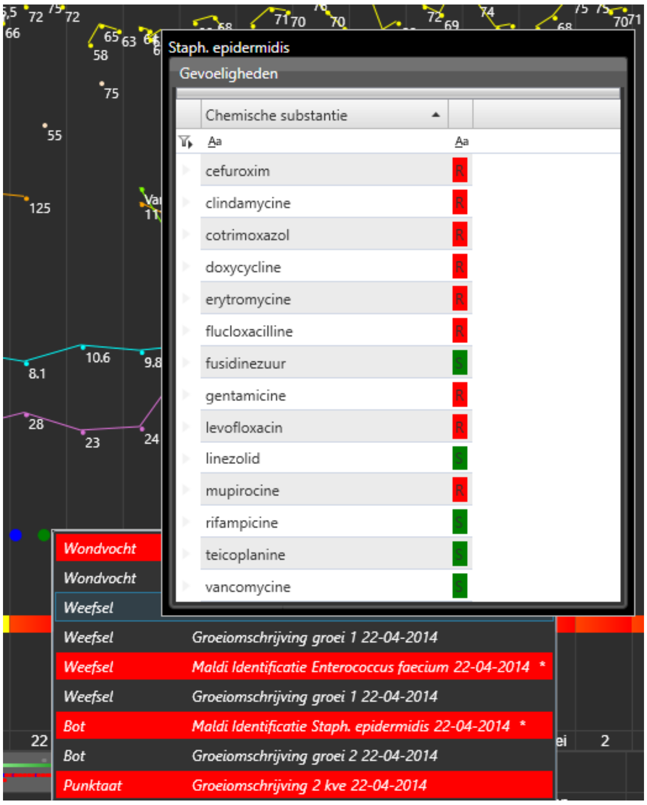

Figure 2: Microorganism details and susceptibility

Scoring and Conflict Resolution

The infection prevention professionals score each patient on the presence or absence of a HAI, including subcategory. They can add notes and nominate a patient for further discussion. If a reviewer has finished reviewing a population the review is closed. When both the first and second reviews have been closed the conflicts between the reviews are automatically selected for further conflict resolution.

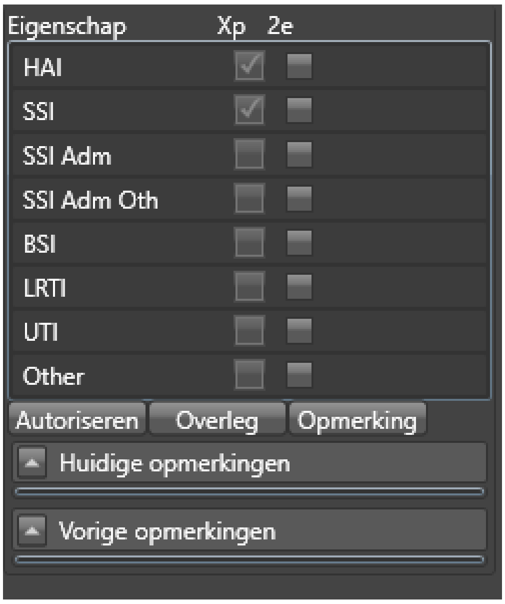

Figure 3: Documenting the results of ICP's assessment

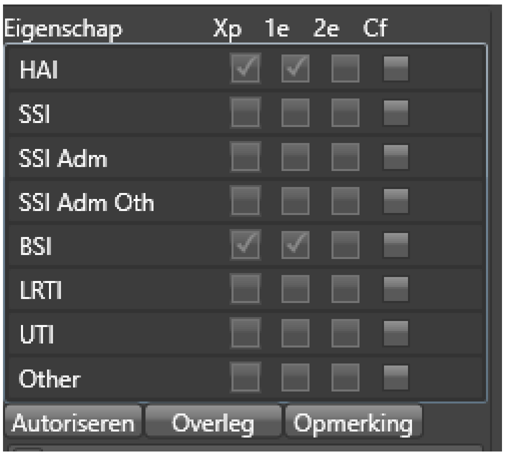

Figure 4: Conflict resolution

The scores of the expert rules, the first and second reviewer are presented together with any notes that the first or second reviewer has made. The reviewers discuss the case and come to a final conclusion. After all the conflicts have been resolved the population can be authorized by the infection control coordinator, making the information available for epidemiological analysis and reporting.
